# HIF-1α activates hypoxia-induced BCL-9 expression in human colorectal cancer cells

**DOI:** 10.18632/oncotarget.8834

**Published:** 2016-04-18

**Authors:** Zhen Tan, Quan Huang, Jia Zang, Shi-Feng Teng, Tian-Rui Chen, Hai-feng Wei, Dian-Wen Song, Tie-Long Liu, Xing-Hai Yang, Chuan-Gang Fu, Zhi-qian Hu, Wang Zhou, Wang-Jun Yan, Jian-Ru Xiao

**Affiliations:** ^1^ Department of Bone Tumor Surgery, Changzheng Hospital, Second Military Medical University, Shanghai, China; ^2^ Department of Colorectal Surgery, Changzheng Hospital, Second Military Medical University, Shanghai, China; ^3^ Department of Colorectal Surgery, Changhai Hospital, Second Military Medical University, Shanghai, China

**Keywords:** B-cell CLL/lymphoma 9 protein, hypoxia inducible factors-1α, hypoxia, colorectal cancer

## Abstract

B-cell CLL/lymphoma 9 protein (BCL-9), a multi-functional co-factor in Wnt signaling, induced carcinogenesis as well as promoting tumor progression, metastasis and chemo-resistance in colorectal cancer (CRC). However, the mechanisms for increased BCL-9 expression in CRC were not well understood. Here, we report that hypoxia, a hallmark of solid tumors, induced BCL-9 mRNA expression in human CRC cells. Analysis of BCL-9 promoter revealed two functional hypoxia-responsive elements (HRE-B and HRE-C) that can be specifically bound with and be transactivated by hypoxia inducible factors (HIF) -1α but not HIF-2α. Consistently, ectopic expression of HIF-1α but not HIF-2α transcriptionally induced BCL-9 expression levels in cells. Knockdown of endogenous HIF-1α but not HIF-2α by siRNA largely abolished the induction of HIF by hypoxia. Furthermore, there was a strong association of HIF-1α expression with BCL-9 expression in human CRC specimens. In summary, results from this study demonstrated that hypoxia induced BCL-9 expression in human CRC cells mainly through HIF-1α, which could be an important underlying mechanism for increased BCL-9 expression in CRC.

## INTRODUCTION

Colorectal cancer (CRC) is the third most common cancer and the fourth most major cause of malignancies death, accounting for ∼1.2 million new cases and ∼600,000 deaths annually worldwide [1]. Several significant efforts have been devoted to identify the etiology of CRC; however, the accurate mechanism of CRC carcinogenesis remains unknown.

Wnt signaling pathway plays critical roles in gene functions during normal to malignant development and transcriptional programs of cell self-renewal [2]. Abnormal activation of Wnt signaling pathway is reported to be associated with a variety of human cancers such as CRC, breast cancer and prostate cancer [3–7]. The great majority (> 90%) of CRC carries mutation of at least one gene in Wnt signaling pathway [8]. β-catenin as a key effector phosphorylates and targets for degradation by Axin complex, which is inhibited in respond to Wnt stimulation. This process flow allows unphosphorylated β-catenin to accumulate and bind with the nuclear TCF/LEF to stimulate the transcription of Wnt target gene, resulting in recruiting chromatin modifying and remodeling a range of transcriptional complex and co-factors [9–11]. Well-established transcriptional complexes and co-factors involving B-cell CLL/lymphoma 9 protein (BCL-9), Pygopus (PYGO) and polymerase-associated factor 1 (PAF1) have been reported to be recruited by β-catenin [9].

BCL-9, one of the crucial counterparts of the nuclear component Legless (Lgs) in Wnt pathway, is highly expressed in human CRC tissues, and the BCL-9/β-catenin complex has been identified as a crucial target for cancer therapy [12]. The molecular role of BCL-9 is currently debated. One function of Lgs/BCL-9 proteins could recruit transcriptional co-factors synergized with β-catenin and its co-factors during the transcription of TCF target genes. Likewise, they could capture nuclear β-catenin to facilitate its recruitment to TCF target genes [9, 13]. However, there are very little studies working on the mechanisms of regulation on BCL-9 and its co-factors related functional. In this study, we focused on the mechanism of regulation on BCL-9 via hypoxia signaling pathway, and hypothesized that BCL-9 in both Wnt and hypoxia signaling plays a vital role on the carcinogenesis of human CRC.

## RESULTS

### Association between BCL-9 expression and clinicopathological parameters in CRC patients

**Figure 1:**
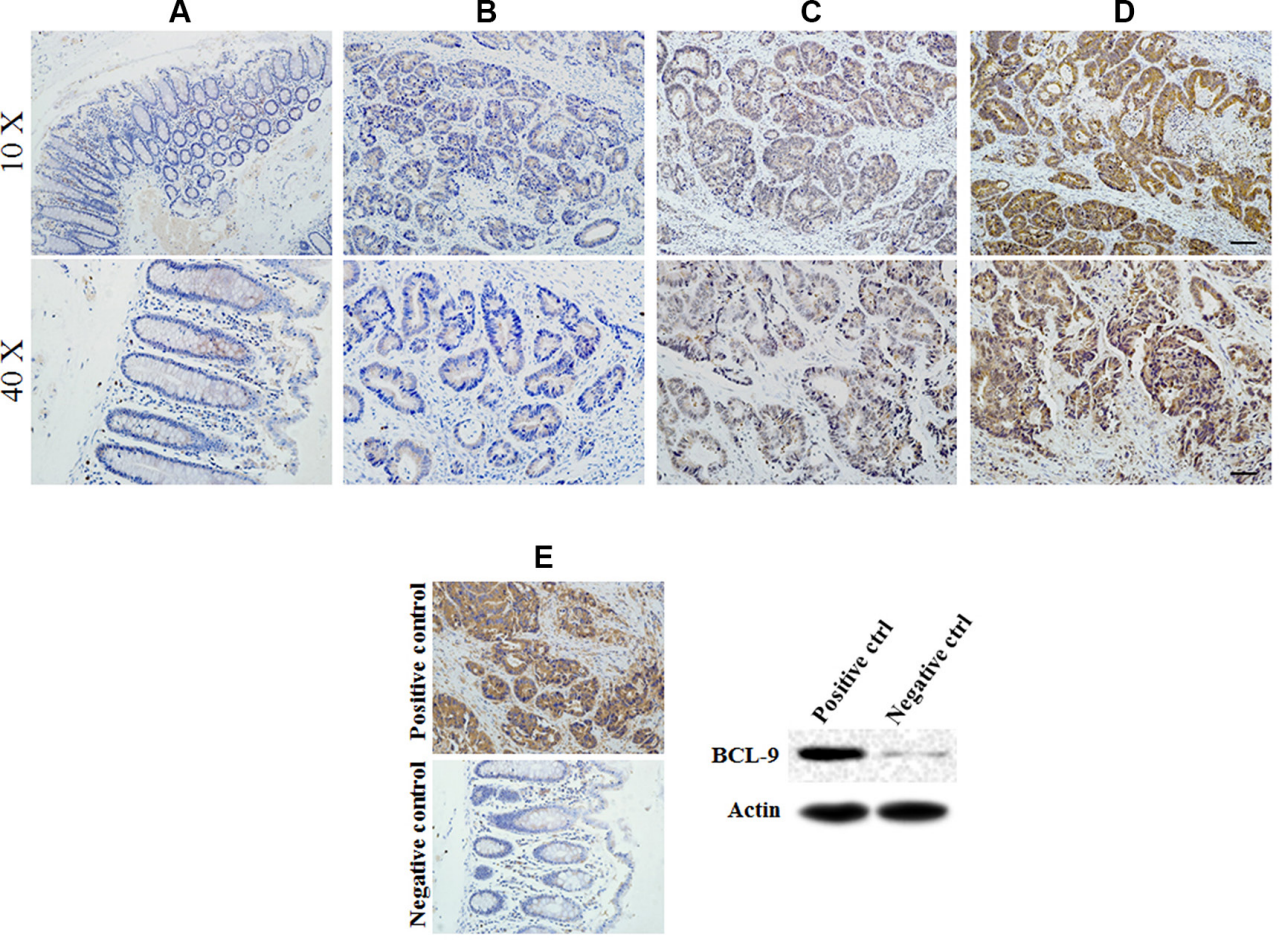
BCL-9 expression in different developing stages of colorectal cancer tissue Representative immunohistochemical stained imaging of BCL-9 expression in different developing stages of colorectal cancer tissue containing 284 cases were shown. Positive cells were stained brown. (**A**) BCL-9 expression in normal mucosa. (**B**) low intensity expression in colorectal cancer cells scored as “−”. (**C**) moderate intensity expression in colorectal cancer cells scored as “+”. (**D**). high intensity expression in colorectal cancer cells scored as “++”. (**E**) Positive and negative control of BCL-9 expression showed the specificity of staining and were validated by Western-blot assays. Scale bar: 40 μm for low magnitude images (10×); 10 μm for high magnitude images (40×).

Expression of BCL-9 showed nuclear staining scored as −, +, ++ in cases examined. Different staining levels of BCL-9 expressed in the cytoplasm of epithelial cells were show in Figure 1. The positive expression of BCL-9 was observed in all stages of CRC samples (13.9% in stage I, 10 of 72 cases; 47.4% in stage II, 63 of 133 cases; 55.8% in stage III, 43 of 77 cases; and 100% in stage IV, 2 of 2 cases).

As our related work described previously, specimen selection for 284 cases were included. Demographic clinicopathological characteristics including gender, age, invasive depth, lymph node metastasis, distant metastasis, AJCC TNM stage and tumor pathological type were summarized in Table 1. BCL-9 expression was associated with AJCC TNM stage (*p* = 0.000), invasive depth (*p* = 0.000) and lymph node metastasis (*p* = 0.000). No significant association was observed in BCL-9 expression compared with distant metastasis and tumor pathological type (both, *p* > 0.05).

**Table 1:** Relationship of BCL-9 expression and clinicopathological parameters in colorectal cancer patients (*n* = 284)

Variable	*n*	BCL-9 expression			*p*-value
		−	+	++	
Gender					0.894*
Male	166	98	48	20	
Female	118	68	37	13	
Age (yr)					0.132*
< 65	161	101	42	18	
≥ 65	123	65	43	15	
Invasion depth					0.000**
T1	3	3	0	0	
T2	82	66	13	3	
T3	195	97	71	27	
T4	4	0	1	3	
Lymph node metastasis					0.000**
N0	205	132	55	18	
N1	71	34	24	13	
N2	8	0	6	2	
Distant metastasis					0.191*
M0	282	166	83	33	
M1	2	0	2	0	
AJCC TNM stage					0.000**
I	72	62	7	3	
II	133	70	47	16	
III	77	34	29	14	
IV	2	0	2	0	
Tumor pathological type					0.527**
Adenocarcinoma	258	148	80	30	
Mucinous adenocarcinoma	15	10	3	2	
Papillary adenocarcinoma	9	7	1	1	
Undifferentiated carcinoma	1	0	1	0	
Signet-ring cell carcinoma	1	0	0	1	

* using Mann-Whitney *U* test;

** using Kruskal-Wallis test.

### Hypoxia induces BCL-9 expression in human CRC cells

**Figure 2:**
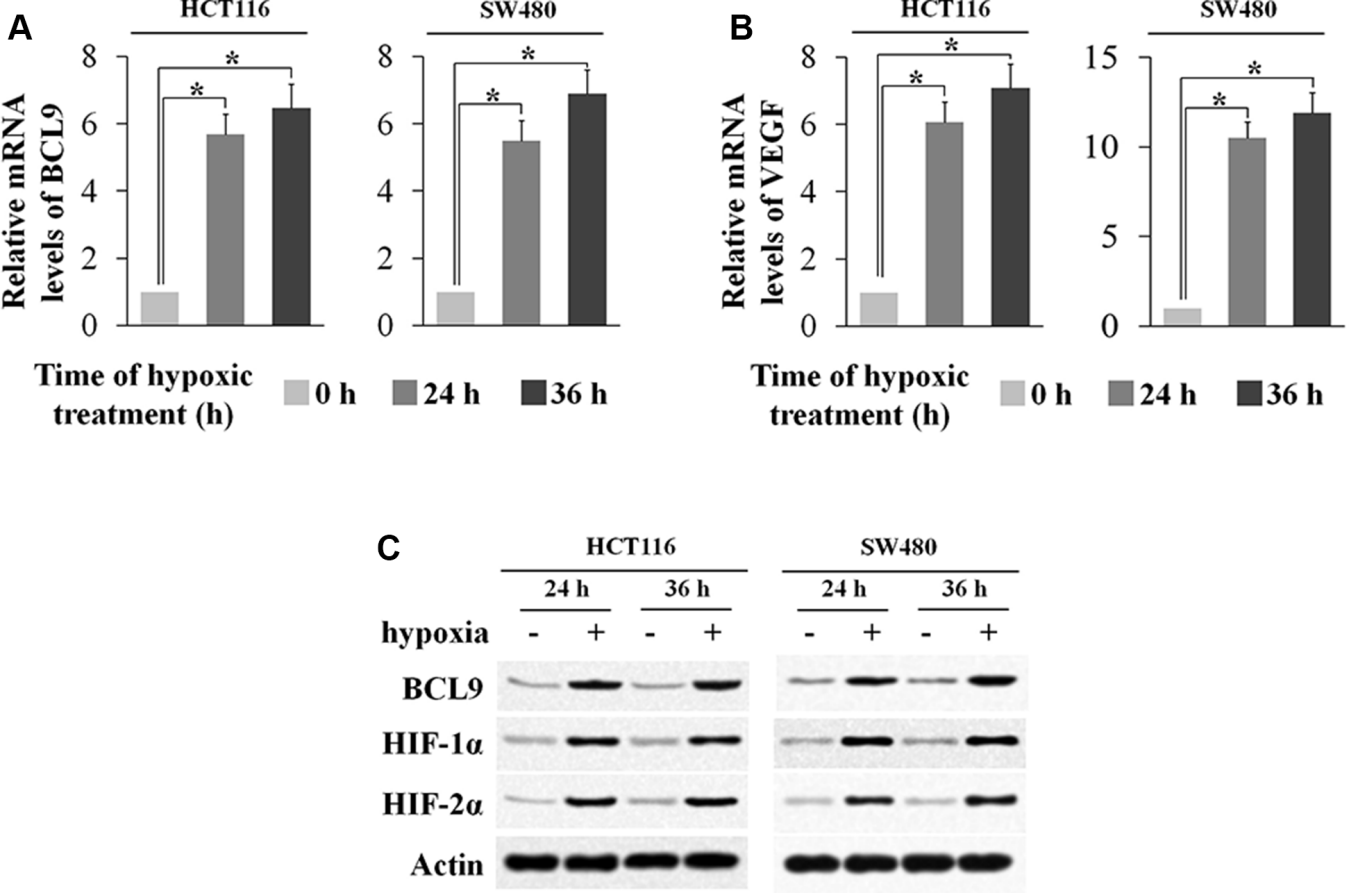
Hypoxia induces BCL-9 expression levels in human colorectal cancer cell lines Human colorectal cancer cell lines SW480 and HCT116 cells were cultured under the hypoxic condition for the indicated time periods. (**A**) The mRNA expression levels of BCL-9 in these cells were determined by Taqman real-time PCR and normalized with actin. (**B**) The mRNA expression levels of VEGF in these cells were determined as a positive control. (**C**) The BCL-9 protein levels were determined by Western-blot assays. Data are presented as mean ± SD (*n* = 3). *p < 0.01, Student's *t*-test.

We have shown that a high percentage of human colorectal cancer specimens display elevated BCL-9 expression levels, but the underlying mechanism for the increased expression of BCL-9 in CRC is unclear. So we introduce the microenvironment Hypoxia, a hallmark of solid tumors including CRC. Interestingly, we found that BCL-9 expression was induced by hypoxia. Human CRC cell lines SW480 and HCT116 cells were treated by hypoxia (0.1% O_2_). The effective induction of hypoxia in cells was confirmed by the increased expression of VEGF, a hypoxia-responsive gene, at mRNA levels and the increased protein levels of HIF-1α and HIF-2α, the major hypoxia inducible factors. Notably, BCL-9 mRNA levels were significantly increased (*p* < 0.01) in both SW480 and HCT116 cells cultured under the hypoxic condition as determined by real-time PCR (Figure 2A–2B). The induction of BCL-9 expression by hypoxia was confirmed at the protein levels in these cells by using Western-blot assay (Figure 2C). These data clearly demonstrated that hypoxia induces BCL-9 expression in human colorectal cancer cells.

### Hypoxia transactivates the BCL-9 promoter region containing hypoxia-responsive elements (HREs)

**Figure 3:**
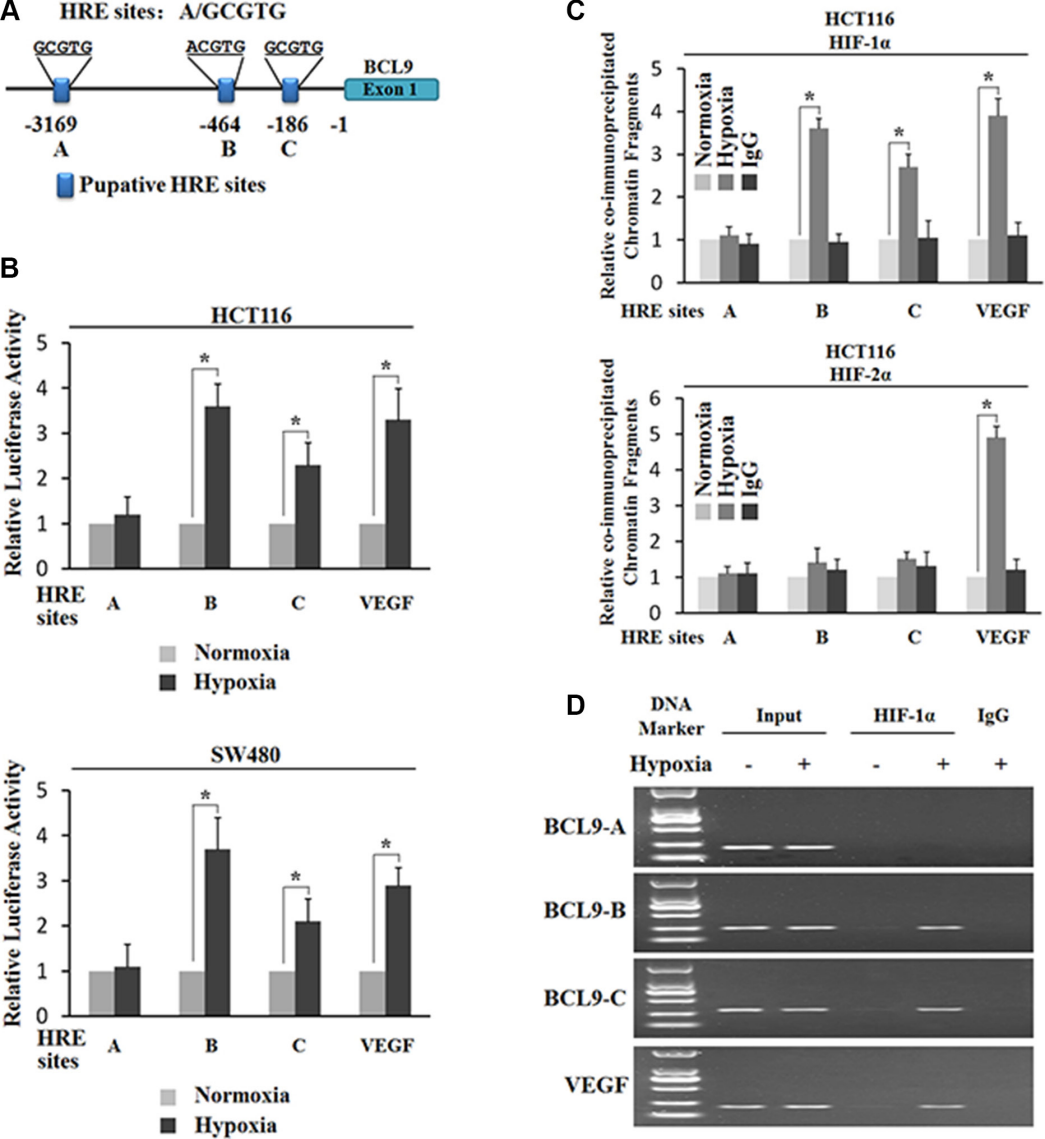
Hypoxia transactivates hypoxia-responsive elements (HREs) in the BCL-9 promoter through HIF-1α (**A**) The human BCL-9 gene contains 3 putative HREs in the promoter region. (**B**) Hypoxia activates the luciferase activity of reporter vectors containing HRE-B or HRE-C sites in the BCL-9 promoter. SW480 and HCT116 cells were transfected with the luciferase reporter vectors, and then subjected to hypoxia treatment for 36 h before measuring luciferase activities. Luciferase reporter vectors containing the HRE site in the VEGF promoter was included as a positive control. (**C**) and (**D**) HIF-1α but not HIF-2α binds to HRE-B and HRE-C sites in the BCL-9 promoter under the hypoxic condition in HCT116 cells as determined by ChIP assays. Cells were cultured under the hypoxic or normoxic conditions for 36 h before assays. The HRE site in the VEGF promoter serves as a positive control. The amount of DNA fragments pulled-down was determined by real-time PCR (C) or conventional PCR (D). Data are presented as mean ± SD (*n* = 3). *p < 0.01 (Student's *t*-test).

The transcriptional response to hypoxia in cells is largely mediated by HIFs. HIF-1α and HIF-2α induce a number of common genes, and at the same time they each have their unique target genes. To investigate whether the transcriptional induction of BCL-9 by hypoxia is activated by HIFs, we searched for the HRE consensus sequence in the promoter region of the BCL-9 gene from 3 kb upstream of transcriptional start site. Three putative HRE sites (HRE-A, HRE-B and HRE-C) were identified in the promoter region of the BCL-9 gene (Figure 3A). To investigate whether these putative HREs account for the hypoxia-mediated induction of BCL-9, the DNA fragments containing one copy of these putative HRE sites were inserted into a pGL2 luciferase reporter plasmid. VEGF is a well-known hypoxia-inducible gene. HIFs physically bind with the HRE in the VEGF promoter and induce VEGF expression levels under hypoxia. The DNA fragment containing the HRE in the VEGF promoter was inserted into a pGL2 luciferase reporter plasmid to serve as a positive control. SW480 and HCT116 cells were transiently transfected with these reporter constructs, and then exposed to hypoxia for 36 h. TK100 plasmids was co-transfected as an internal standard to normalize transfection efficiency. As shown in Figure 3B, hypoxia clearly enhanced luciferase expression levels of the reporter plasmids containing the HRE from the VEGF promoter, the HRE-B and HRE-C from the BCL-9 promoter, but did not have a clear effect on HRE-A form the BCL-9 promoter in both cells. These results indicate that hypoxia transactivates the BCL-9 promoter containing functional HREs (HRE-B and HRE-C).

### HIF-1α instead of HIF-2α binds to HRE-B and HRE-C in the BCL-9 promoter under the hypoxic condition

To further investigate which HIF (HIF-1α or HIF-2α) activates the hypoxia-induced BCL-9 expression, chromatin immunoprecipitation (ChIP) assays were employed to determine whether HIF-1α and HIF-2α physically bind to HREs, especially HRE-B and HRE-C in the BCL-9 promoter. Both SW480 and HCT116 cells were cultured under the normoxic or hypoxic conditions for 36 h, and the ChIP assays were performed using an antibody against HIF-1α or HIF-2α. The degree of pull-down of chromatin fragments was determined by quantitative real-time PCR. As shown in Figure 3C, both HIF-1α and HIF-2α bound with the chromatin fragments containing the HRE from the VEGF promoter under the hypoxic but not normoxic conditions. However, no clear immunoprecipitation of the chromatin fragments containing HRE-A, HRE-B, and HRE-C by the antibody against HIF-2α was observed in both SW480 and HCT116 cells under either the hypoxic or normoxic conditions (Figure 3C). Interestingly, the chromatin fragments containing HRE-B and HRE-C but not HRE-A were pulled down by the antibody against HIF-1α in both SW480 and HCT116 cells under the hypoxic condition (Figure 3C). Furthermore, these chromatin fragments were not co-immunoprecipitated with the HIF-1α antibody in cells under the normoxic condition (Figure 3C). The specific pull-down of chromatin fragment containing HRE-B and HRE-C but not HRE-A by the HIF-1α antibody under the hypoxic condition was also confirmed by conventional PCR followed by agarose gel electrophoresis (Figure 3D). These results demonstrated that HIF-1α but not HIF-2α binds with HRE-B and HRE-C in the BCL-9 promoter under the hypoxic condition, which may stimulate the hypoxia-induced BCL-9 expression.

### HIF-1α transcriptionally stimulates BCL-9 expression

**Figure 4:**
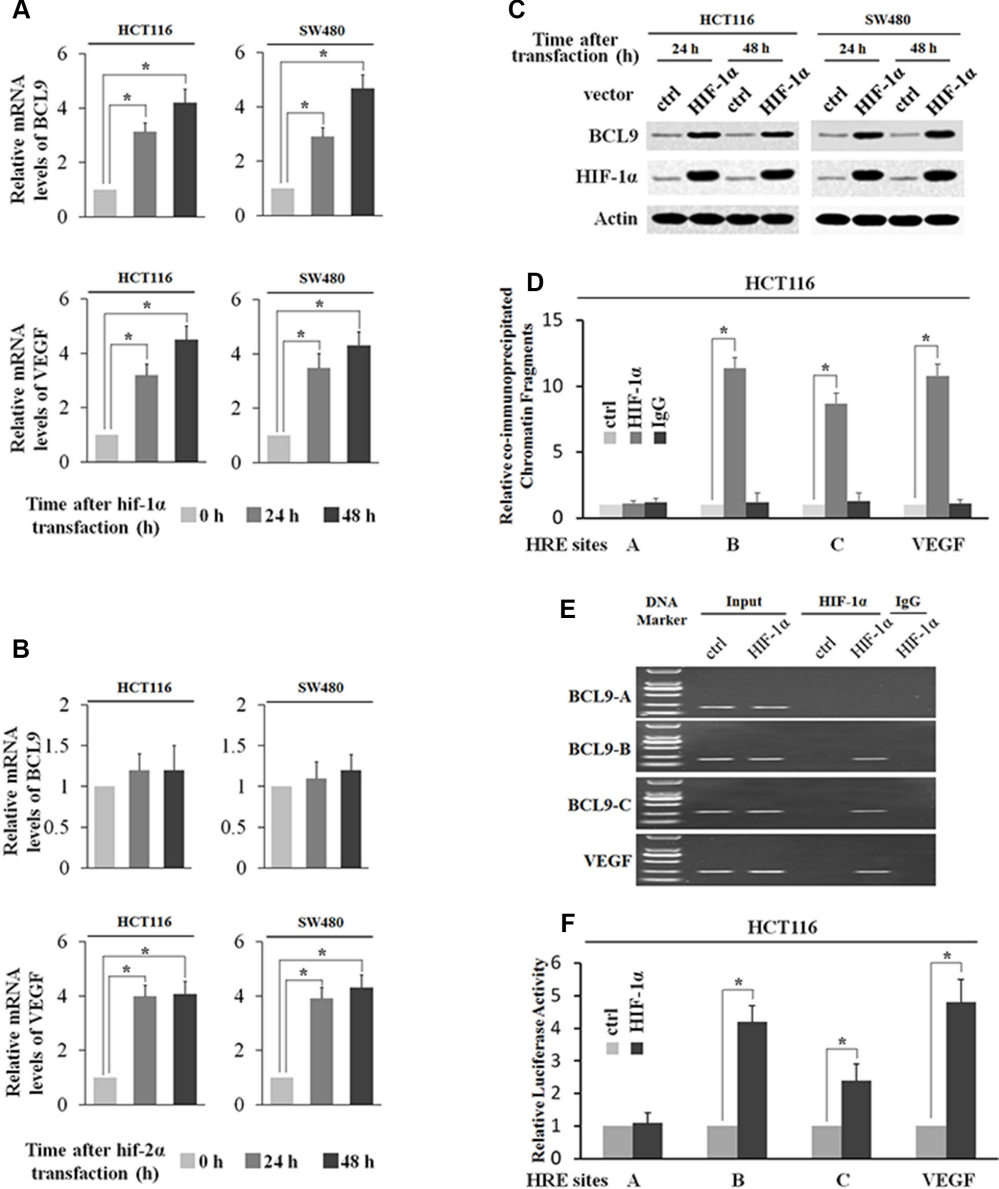
HIF-1α transcriptionally stimulates BCL-9 expression (**A**) Ectopic HIF-1α expression increases BCL-9 and VEGF mRNA expression levels in SW480 and HCT116 cells. The mRNA expression levels of BCL-9 and VEGF were determined by Taqman real-time PCR and normalized with actin. (**B**) Ectopic HIF-2α expression increases VEGF mRNA expression levels but has no effect on mRNA BCL-9 expression levels in SW480 and HCT116 cells. (**C**) Ectopic HIF-1α expression increases BCL-9 protein levels in SW480 and HCT116 cells as determined by Western-blot assays. (**D**) and (**E**) HIF-1α binds to HRE-B and HRE-C sites in the BCL-9 promoter in HCT116 cells transfected with HIF-1α expression plasmids as determined by ChIP assays. The amount of DNA fragments pulled-down was determined by real-time PCR (D) or conventional PCR (E). The HRE site in the VEGF promoter serves as a positive control. (**F**) HIF-1α activates luciferase activity of reporter vectors containing HRE-B or HRE-C sites in the BCL-9 promoter in HCT116 cells transfected with HIF-1α expression plasmids. Luciferase reporter vectors containing the HRE site in the VEGF promoter was included as a positive control. Data are presented as mean ± SD (*n* = 3). *p < 0.01 (Student's *t*-test).

To directly investigate whether HIF-1α but not HIF-2α transcriptionally induces BCL-9 expression levels, SW480 and HCT116 cells were transfected with the plasmids expressing HIF-1α (pcDNA3-Flag-HIF-1α) and HIF-2α (pcDNA3-Flag-HIF-2α) respectively. The ectopic expression of either HIF-1α or HIF-2α clearly induced VEGF mRNA expression (Figure 4A and 4B). Ectopic HIF-1α expression clearly increased BCL-9 expression at both mRNA and protein levels as determined by Real-time PCR and Western-blot assays respectively (Figure 4A and 4C). However, ectopic HIF-2α expression had no apparent effect on BCL-9 expression (Figure 4B). Furthermore, the degree of pull-down of chromatin fragments containing HREs in the BCL-9 promoter by HIF-1α was determined by ChIP assays in HCT116 cells with ectopic HIF-1α expression. As shown in Figure 4D–4E, chromatin fragments containing HRE-B and HRE-C but not HRE-A was co-immunoprecipitated with the Flag antibody in cells with ectopic HIF-1α expression as determined by both real-time PCR and conventional PCR assays. These observations clearly showed that HIF-1α bound with HRE-B and HRE-C in the BCL-9 promoter, which is consistent with the ChIP results obtained in cells under the hypoxic condition. We further investigated whether HIF-1α directly transactivates HREs in the BCL-9 promoter. HCT116 cells were co-transfected with an expression plasmid of HIF-1α and the pGL2 reporter plasmids containing HREs in the BCL-9 promoter or the HRE in the VEGF promoter along with TK100 plasmids. Ectopic HIF-1α expression increased the luciferase activity of the reporter plasmids containing the HRE in the VEGF promoter (Figure 4F). Notably, ectopic HIF-1α expression clearly transactivated the reporter plasmids containing HRE-B and HRE-C from the BCL-9 promoter, but had no apparent effect on the reporter plasmids containing HRE-A from the BCL-9 promoter (Figure 4F). Collectively, these results clearly showed that HIF-1α binds with and transactivates HRE-B and HRE-C in the BCL-9 promoter to directly induce BCL-9 expression in cells.

### HIF-1α activates the induction of BCL-9 expression by hypoxia

**Figure 5:**
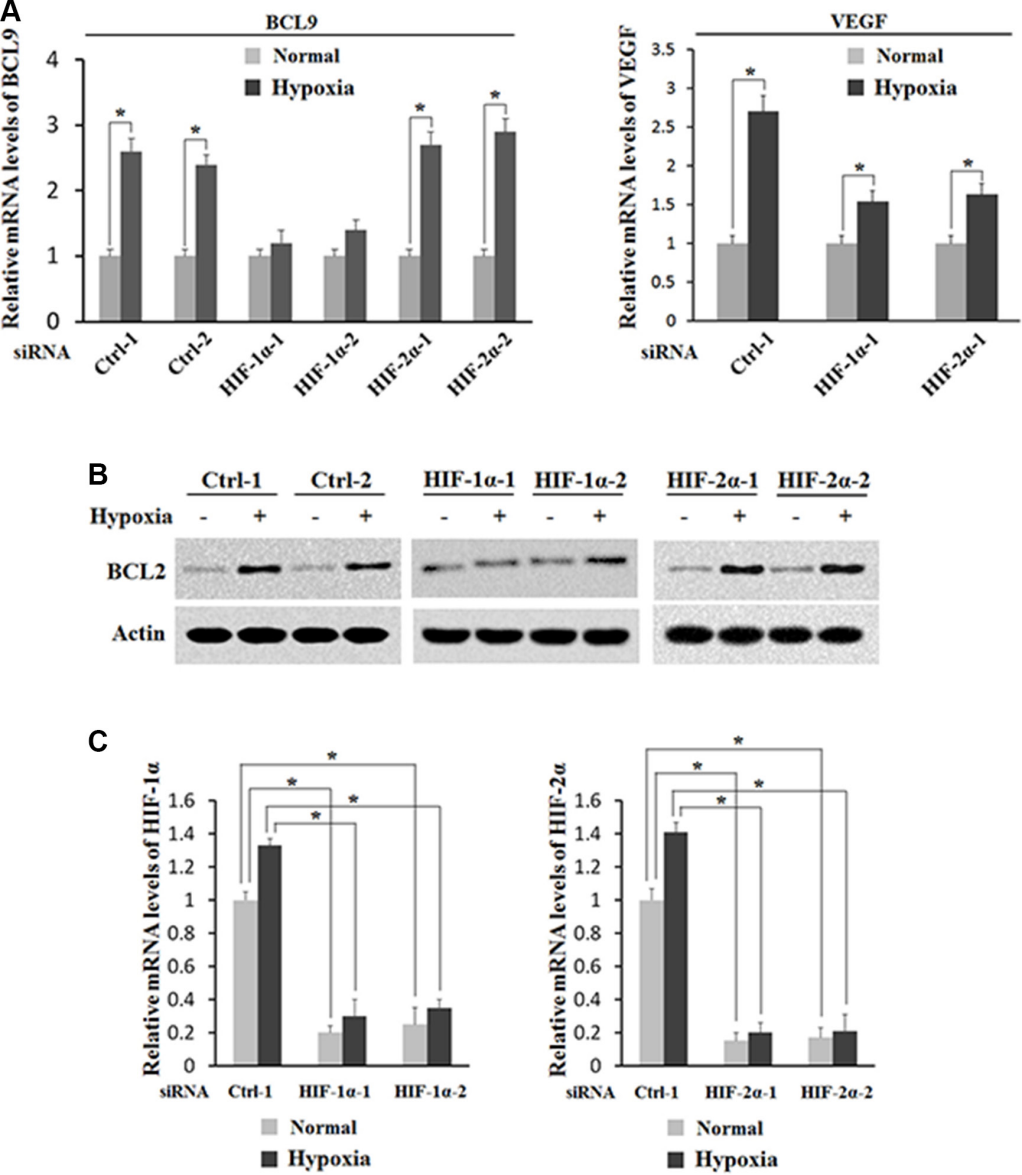
HIF-1α activates the induction of BCL-9 expression by hypoxia Knockdown of endogenous HIF-1α but not HIF-2α largely abolishes the induction of BCL-9 expression by hypoxia in HCT116 cells. Cells with knockdown of endogenous HIF-1α, HIF-2α by siRNA oligos or transfected with control siRNA were treated with hypoxia for 36 h. Two different siRNA oligos against HIF-1α and HIF-2α, respectively, were used, and similar results were obtained. (**A**) The mRNA expression levels of BCL-9 (left panel) and VEGF (right panel) were determined by Taqman real-time PCR and normalized with actin. (**B**) The BCL-9 protein levels were determined by Western-blot assays. (**C**) The knockdown of HIF-2α (left panel) and HIF-1α (right panel) in cells was conformed at the mRNA level by Taqman real-time PCR and normalized with actin. Data are presented as mean ± SD (*n* = 3). *p < 0.01 (Student's *t*-test).

To investigate whether HIF-1α but not HIF-2α activates the induction of BCL-9 expression by hypoxia, the effect of hypoxia on BCL-9 expression levels was determined in HCT116 cells with knock-down of endogenous HIF-1α or HIF-2α by siRNA. As shown in Figure 5A and 5B, knock-down of endogenous HIF-1α largely abolished hypoxia-induced BCL-9 expression in HCT116 cells as determined at both mRNA and protein levels by Real-time PCR and Western-blot assays respectively. In contrary, knockdown of endogenous HIF-2α by siRNA had no apparent effect on hypoxia-induced BCL-9 expression (Figure 5A and 5B). It has been shown that VEGF is a common target of HIF-1α and HIF-2α. Indeed, knockdown of either HIF-1α or HIF-2α can all inhibit hypoxia-induced VEGF expression (Figure 5A). The effective knockdown of HIF-1α and HIF-2α was confirmed at the mRNA level by Real-time PCR assays (Figure 5C). These results clearly demonstrated that hypoxia induces BCL-9 expression mainly through HIF-1α.

### HIF-1α expression is associated with BCL-9 expression in human CRC specimens

**Figure 6:**
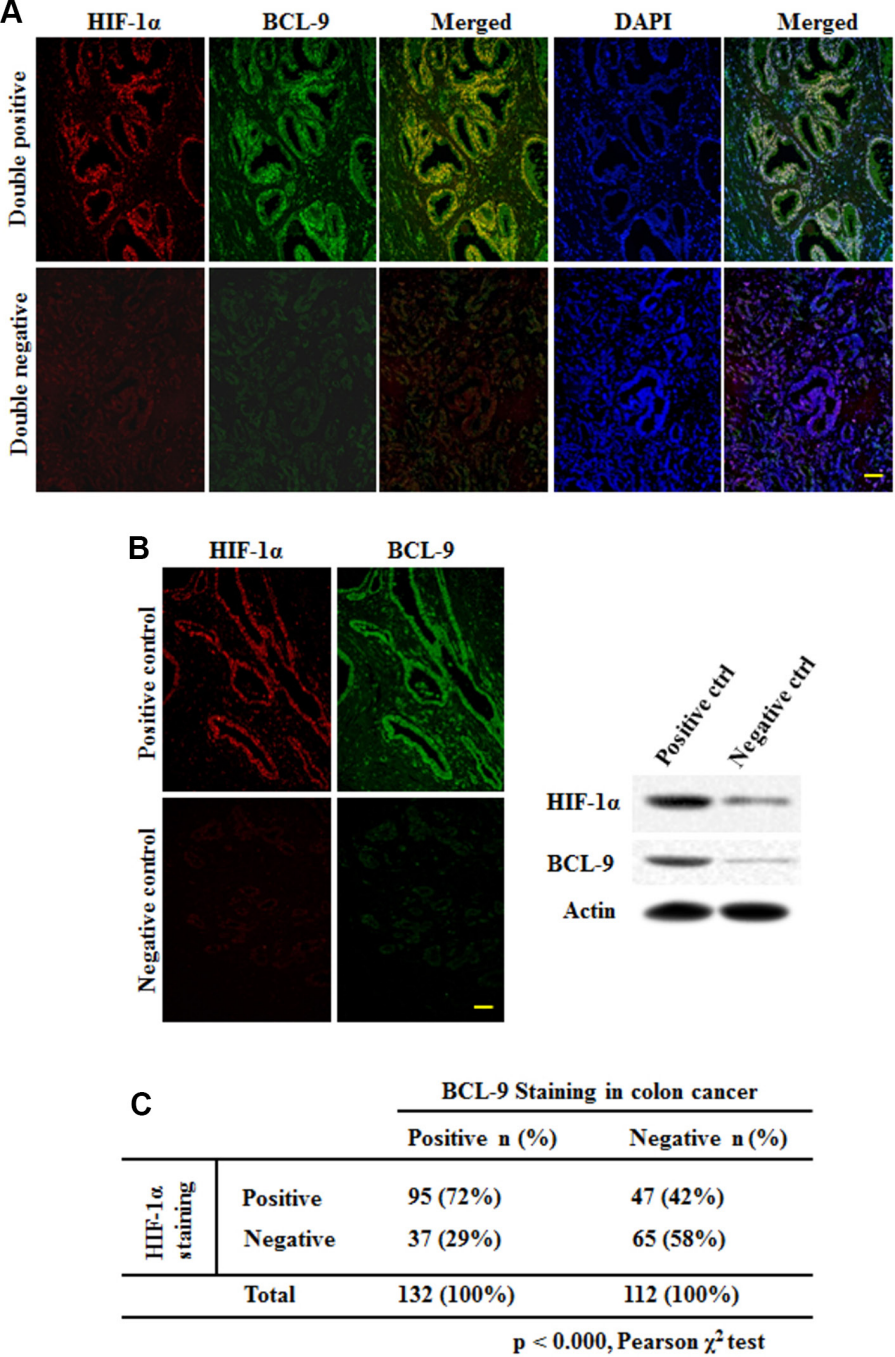
HIF-1α expression is associated with BCL-9 expression in human colorectal cancer specimens HIF-1α and BCL-9 protein levels were examined on specimens of 244 cases of human colorectal cancer by the double immunofluorescence (IF) co-localization. (**A**) Representative IF staining results for HIF-1α (upper panels) and BCL-9 (lower panels) are shown. Positive HIF-1α or BCL-9 staining: > 25% cells stained with HIF-1α or BCL-9, respectively. (**B**) Positive and negative control of BCL-9 expression showed the specificity of staining and were validated by Western-blot assays. Scale bar: bar = 10 μm. (**C**) HIF-1α expression is associated with BCL-9 expression in human colorectal cancer (*p* < 0.000, Pearson chi-square test).

To investigate whether hypoxia and HIF-1α contribute to the increased expression of BCL-9 in human CRC samples, the expression levels of BCL-9 and HIF-1α were determined by the double immunofluorescence (IF) co-localization and stained on slides comprising 244 human CRC samples. The representative IF images of BCL-9 and HIF-1α were shown in Figure 6A. A significant portion of CRC samples (54%; 132 out of 244 cases) showed positive BCL-9 staining. There is also a significant portion of CRC samples (58%; 142 out of 244 cases) showed positive HIF-1α staining. Notably, HIF-1α expression is strongly associated with BCL-9 expression (Figure 6B) (*p* < 0.000, Pearson chi-square test). HIF-1α positive staining was observed in 72% of cases with BCL-9 positive staining (95 of 132 cases), but in 42% of cases with BCL-9 negative staining (47 of 112 cases). These results strongly suggested that the transcriptional induction of BCL-9 by hypoxia and HIF-1α is an important mechanism accounting for the BCL-9 expression in human CRC.

## DISCUSSION

Hypoxia-signaling pathway as a classic hallmark of solid tumor was widely concerned. Due to restricted blood flow, tumor cells experienced hypoxic condition that inhibited cell proliferation and altered energy metabolism from oxidative phosphorylation to glycolysis pathway, resulting in modifications of hypoxia-inducible genes expressions [14, 15]. These are mediated by the hypoxia inducible factors (HIFs), which contained three HIF-α (HIF-1α, HIF-2α and HIF-3α) and two HIF-β (HIF-1β and HIF-2β) [16]. Compared to the Wnt signaling, the hypoxia signaling does not require secreted or transmembranous proteins but does sense oxygen concentrations instead. Once activated under hypoxia, HIF-α are facilitated and dimerized with HIF-1 β; this complex binds to HRE and triggers transcription of target genes such as vascular endothelial growth factor (VEGF), leading to stimulation of carcinogenesis [17, 18]. Since there was a crosstalk with different signaling pathways like hypoxia, Hedgehog and Wnt pathway, it was suggested that carcinogenesis is often dependent on at least two pathways [24]. In our study, one of the first evidence for crosstalk between Wnt and hypoxia signaling emerged when hypoxia leaded to upregulation of the key Wnt co-activatior BCL-9 in HIF-1α/2α-correlated manners, suggesting that hypoxia pathway might promote oncogenic Wnt signaling by recruiting HIF-α/BCL-9 interaction.

HIF-1α and HIF-2α are often overexpressed in solid tumors and tumor-derived cell lines [19]. Although sometimes both these proteins share similar properties mainly via transcriptional regulation of common target genes, they each target own genes contributing to specific characteristics [20, 21]. In addition, the different selection of HIF-1α and HIF-2α target genes can be contributed by interacting with different transcription factors and/or chromatin context [22, 23]. Base on this characteristic of HIFs, in our study, we found that BCL-9 promoter contains 3 putative HRE sites including HRE-A, HRE-B and HRE-C. Meanwhile ChIP assays showed that HIF-1α but not HIF-2α bound with HRE-B and HRE-C but not HRE-A in the BCL-9 promoter under hypoxic condition. Moreover, knockdown of HIF-1α but not HIF-2α largely abolished hypoxia-induced BCL-9 expression. These results clearly demonstrate that BCL-9 is a target gene for HIF-1α but not HIF-2α. Intriguingly, transfection and knockdown of either HIF-1α or HIF-2α can respectively promote and inhibit hypoxia-induced VEGF expression, suggesting that VEGF is a common identical target gene for hypoxia signaling pathway.

In summary, this study demonstrated that BCL-9 expression could be induced by hypoxia in human CRC cells; BCL-9 induction under hypoxic condition is predominantly activated by HIF-1α. Given the critical role of BCL-9 in Wnt signaling pathway promoting carcinogenesis and chemo-resistance, the specific regulation of BCL-9 expression by HIF-1α is considered to be a underlying crosstalk between Wnt signaling and hypoxia signaling pathway, providing foundation for further study to develop new target of potential therapeutic strategies by blocking HIF-1α/BCL-9 signaling related expression in CRC.

## MATERIALS AND METHODS

### Patients and samples

A total of CRC specimens were obtained from patients who underwent colectomy at the Department of Surgery, Changzheng Hospital, Second Millitary Medical University, Shanghai, China, from January 2012 to October 2015. None of the patients in our study received neoadjuvant chemotherapy. Information on patient gender, age, invasive depth, lymph node metastasis, distant metastasis, AJCC TNM stage and tumor pathological type were summarized in Table 1. The tumor pathological types of the samples were assessed by the experienced colorectal pathologists. Informed consent forms were signed from all patients and the research protocol was approved by the Ethics Committee.

### IHC and IFC staining assays

Tumor tissues or other samples were fixed with 4% paraformaldehyde and were then dehydrated through a graded series of ethanol, embedded in paraffin and sectioned at 5 μm. Immunohistochemical staining for BCL-9 was carried out using standard histological procedures described in the manual for Histostain-Plus IHC Kit, DAB (Phygene). BCL-9 staining was scored in accordance by two investigators with previous protocols as negative (−), weak positive (+), or strong (++). Scoring was performed blindly with respect to the histologic grade of CRC specimens.

Immunofluorescence staining was performed with primary and secondary antibodies diluted in 10% BSA and nucleus was stained by DAPI (Sigma). All fluorescent secondary antibodies were used at a dilution of 1:200 for 30 min (invitrogen). Quantification of IF was performed using NIH ImageJ.

### Cell culture and cell treatment

Human CRC cell lines HCT116 and SW480 were obtained from ATCC. Cells were cultured in DMEM supplemented with 10% fetal bovine serum (Sigma). For hypoxic treatment, cells at 50–60% confluence were incubated in a hypoxia chamber (0.1% O_2_, STEMCELL). HIF-1α expression plasmids (pcDNA3-Flag-HIF-2α) and HIF-2α expression plasmids (pcDNA3-Flag-HIF-1α) were obtained from GENEwiz^™^. For siRNA knockdown, two different siRNA oligos against HIF-1α and HIF-2α were purchased from Ribobio^™^. siRNA targeting HIF-1α: siRNA-1: 5-CAG ACU UUA UGU UCA UAG UUC UUC CUC-3; siRNA-2: 5-CUU CCA CAA CUA CAU AGG GUA UUG UUU-3. siRNA targeting HIF-2α: siRNA-1: 5-AUA CAG UUA UAA UGU UGU CAG UAG GAA-3; siRNA-2: 5-UCA UUG AAA UCC GUC UGG GUA CUG CAU-3. Expression plasmids and siRNA oligos were transfected into cells using X-treme GENE HP (Roche).

### Real-time PCR

Total RNA was isolated by using RNAiso Plus (TAKARA) following the manufacturer's instruction. RNA was reverse transcribed into cDNA by using the Taqman Reverse Transcription Reagents kit (Applied Biosystems) with random hexamers. Human BCL-9, VEGF, HIF-1α, HIF-2α and actin mRNA levels were determined in 7900HT Fast Real-time PCR system (Life Technology Corporation, USA). All primers were purchased from Applied Biosystems. Real-time PCR was done in triplicate with TaqMan PCR mixture (Applied Biosystems). The expression of genes was normalized to the actin gene.

### Western-blot assays

Standard Western-blot assays were used to analyze the levels of protein. Antibodies against HIF-1α (sc-13515, Santa Cruz Biotechnology, 1:500), anti-HIF-2α (sc-13596, Santa Cruz Biotechnology, 1:500), anti-BCL-9 (ab37305, Abcam, 1:500 dilution) and anti–β-actin (A5441, Sigma) antibodies were used in this study.

### Construction of reporter plasmids

The fragments containing the potential HRE sites (5-G/ACGTG-3) identified from the BCL-9 promoter (HRE-A: −4087 bp∼–3016 bp; HRE-B: −1078 bp∼–323 bp; HRE-C: −313 bp∼822 bp) and VEGF promoter (from −1080 bp to −874 bp upstream of transcription initiation site) were cloned (For HRE A, forward primer: 5-ACC TAT TAT GAG CCT GTG-3, reverse primer: 5-GAG CCT TGC TAT CTG AAC-3; for HRE B, forward primer: 5-GTC GGG ACA GAC AGT TCA-3, reverse primer: 5-GAG ACA GGA AAA AGC CCA-3; for HRE C, forward primer: 5-CAG TGC AGC AGC AAC TAG-3, reverse primer: 5-GTG GTA AGG ATC GGG GTG-3) into pGL2-reporter Luciferase Reporter Vector (Promega) at KpnI-XhoI restriction sites using In-Fusion cloning Kit (TAKARA). All constructs were confirmed by DNA sequencing.

### Luciferase activity assay

To study whether hypoxia transactivates the pGL2-reporter plasmids described above, cells were transiently transfected with the pGL2-reporter plasmids containing one copy of each potential HRE sites together with pRL-null (Promega) as an internal control to normalize transfection by using X-tremeGENE HP (Roche). Cells were then subjected to hypoxia treatment for 48 h. To study the transactivation activity of HIF-1α on pGL2-reporter plasmids, cells were co-transfected with pGL2-reporter plasmids together with HIF-1α expression plasmids. The luciferase activity was measured by using the Dual Luciferase assay kit (Promega) and normalized with the internal standard.

### ChIP assays

ChIP assays were performed by using a ChIP assay kit (Millipore) in accordance with the instructions of the manufacturer. In brief, cells cultured under the hypoxic or normoxic conditions or transfected with HIF-2α expression plasmids were subjected to ChIP assays with anti-HIF-1α or anti-HIF-2α antibodies. Normal IgG was used as a control for nonspecific binding of genomic DNA. DNA fragments pulled-down by antibodies were recovered and subjected to real-time PCR and conventional PCR by using the PCR primers (For HRE A, forward primer: 5-AGA TGG TCT CCG TCT CCT-3, reverse primer: 5-GAG CCT TGC TAT CTG AAC-3; for HRE B, forward primer: 5-CTTATCTCCCTACTCCCC-3, reverse primer: 5-GAG ACA GGA AAA AGC CCA-3; for HRE C, forward primer: 5-CAG TGC AGC AGC AAC TAG-3, reverse primer: 5-GAC AAG CCA CAA ACA AGA C-3, for VEGF promoter, forward primer: 5-CCT CAG TTC CCT GGC AAC ATC TG-3, reverse primer: 5-GAA GAA TTT GGC ACC AAG TTT G-3).

### Statistical analysis

Data was expressed as mean ± SD. The association between HIF-1α expression levels and BCL-9 expression levels examined by IF were analyzed by Pearson chi-square test. Associations between BCL-9 expression examined by IHC and clinicopathological variables were analyzed by non-paramentric analysis: Mann Whitney *U* test for 2 samples and Kruskal Wallis *H* test for more than 2 samples. All other *p* values were obtained by using two-tailed Student's *t*-test. Values of *p* < 0.05 were considered to be significant.
